# Microwave-Assisted Extraction of Phenolic Compounds from Spent Coffee Grounds. Process Optimization Applying Design of Experiments

**DOI:** 10.3390/molecules26237320

**Published:** 2021-12-02

**Authors:** José P. Coelho, Maria P. Robalo, Stanislava Boyadzhieva, Roumiana P. Stateva

**Affiliations:** 1 Instituto Superior de Engenharia de Lisboa, Instituto Politécnico de Lisboa, Rua Conselheiro Emídio Navarro 1, 1959-007 Lisboa, Portugal; mprobalo@deq.isel.ipl.pt; 2 Centro de Química Estrutural, Instituto Superior Técnico, Universidade de Lisboa, Av. Rovisco Pais 1, 1049-001 Lisboa, Portugal; 3 Institute of Chemical Engineering, Bulgarian Academy of Sciences, Academic Georgi Bontchev str., 1113 Sofia, Bulgaria; maleic@abv.bg

**Keywords:** microwave extraction, green technology, spent coffee grounds, design of experiments, total polyphenols content

## Abstract

In this study, sustainable technology microwave-assisted extraction (MAE) in association with green solvents was applied to recover phenolic compounds from spent coffee grounds (SCGs). A design of experiments (DOE) was used for process optimization. Initially, a 2^4−1^ two level Fractional Factorial Design was used and ratios “solvent to solute” and “ethanol to water” were identified as the significant experimental factors. Consequently, Central Composite Design (CCD) was applied to analyze the effects of the significant variables on the response yield, total polyphenols content (TPC), and antioxidant activity (AA) by the DPPH assay method, and quadratic surfaces to optimize those responses were generated. The values of the significant factors of 16.7 (solvent/solute) and 68.9% (ethanol/water) were optimized simultaneously the yield (%) at 6.98 ± 0.27, TPC (mg GAE/g) at 117.7 ± 6.1, and AA (µmol TE/g) at 143.8 ± 8.6 and were in excellent agreement with those predicted from the CCD model. The variations of the compositions of the lipids, caffeine, pentacyclic diterpenes, and FAME as a function of the dominant factor % ethanol in the solvent mixture were analyzed by applying NMR and GC-FID, and the results obtained confirmed their determinative significance.

## 1. Introduction

Coffee is one of the most used plants on Earth; the world coffee production in 2019 to 2020 was estimated at 169.34 million 60-kg bags [1]. Taking into consideration that, from each kg of coffee, 0.91 kg of solid waste is produced, the storage of those huge quantities of spent coffee grains, SCGs, is a critical environmental problem that should be resolved by applying innovative approaches based on the principles of circular economy. Within those concepts, the design and implementation of one feed, multiproduct biorefineries focused on the sustainable use and valorization of SCG waste biomass are a promising alternative. SCG biorefinery should integrate predominantly mild, advanced processes and techniques targeting the clean, safe production of high-value bioactives with enhanced properties and a wide spectrum of applications in the pharmaceutical, nutraceuticals, and cosmetic industries, as well as in the development of functional foods [2,3,4].

From this perspective, among the available techniques for biological and biomass matrix valorization, microwave-assisted extraction (MAE) has proven to be one of the most promising. It is considered an advanced and viable alternative to conventional and other green extraction techniques (e.g., supercritical and subcritical fluid extraction, pressurized liquid extractions, and accelerated fluid and ultrasound-assisted extraction) that has important advantages, among which are controllable and effective heating, faster energy transfer, the reduction of extraction time and use of solvents, higher selectivity, and enhanced yield [5,6,7,8,9,10]. The efficient and rapid heating realized by MAE is related to the dielectric properties of the samples. Dielectric heating rapidly increases the temperatures of the sample matrices volumetrically, significantly reducing the heating time [11]. In addition, MAE in a closed system allows controlled temperature and pressure conditions while preventing the loss of volatile substances and extraction solvent, unlike the open system processes.

Several factors influence the efficiency of the MAE process, namely: the nature of the matrix, power of microwave irradiation, temperature and time of extraction, solvent(s) choice, and solvent/solid ratio, although the influence of the latter could be minimized by applying a system with agitation in the extraction process [5,6,8].

In the literature, until the present, the application of MAE to the extraction of compounds with antioxidant properties, e.g., polyphenols, flavonoids, and others, from SCGs was performed using different microwave equipment and operating conditions. Thus, to recover extracts enriched in the targeted compounds, different irradiation powers, times, and solvents of extraction were applied [12,13,14,15,16]. Some researchers studied the extraction of oil from SCGs, applying an advanced microwave process, and compared its efficiency in terms of yield with a conventional Soxhlet extraction [17]. Other investigators examined the kinetics of antioxidants recovery from SCGs, with particular attention to the influence of the initial thermal ramp on the total polyphenol concentration, total solids, and antiradical power of the final product [18]. The same authors applied the design of experiments (DOE) and response surface modeling (RSM) to determine the optimum operating parameters of the MAE of SCGs, namely solvent composition and temperature range.

Another group investigated the recovery of antioxidants from spent filter coffee, applying ethanol-influenced MAE [12]. RSM was used to analyze the impact of the ethanol concentration and time of microwave radiation on the extraction yield, total polyphenol content (TPC), DPPH radical inhibition activity, and ferric-reducing ability (FRAP). Additionally, the same authors examined the recovery of antioxidants from spent espresso coffee grounds by MAE and again applied RSM to determine the optimum values of the extraction time, liquid-to-solid ratio, and microwave power [15].

The aim of our work was twofold: (i) to study the MAE of phenolic compounds from SCGs by applying design of experiments and the (ii) identification and quantification of the extracts according to the lipidic and fatty acid profiles. To fulfill the first objective, unlike previous studies, two different design of experiment methods were combined and applied in the optimization of the process operating parameters. Hence, initially, a Fractional Factorial Design (FFD) and, subsequently, a Central Composite Design (CCD) were employed to generate a mathematical model that represented the viable interrelationships among four independent parameters: (i) ratio of ethanol to water in the solvent mixture, (ii) microwave irradiation power, (iii) irradiation time, and (iv) solvent to SCG ratios. As response functions, the yield, TPC, and antioxidant activity (AA) by the DPPH assay method were used to analyze the quality of the extracts.

To accomplish the second objective, the composition of certain extracts was analyzed by NMR, while the fatty acid profile was determined by GC-FID. Thus, it was possible to understand the variation of triacylglycerols (TAGS), 1,2-diacylglycerols (1,2-DAGS), and caffeine composition, as well as that of pentacyclic diterpenes of the kaurene family cafestol, 16-O-methylcafestol, and kahweol, as a function of the % of ethanol in the solvent mixture. The fatty acid composition was corroborated by the FAME analyses of the extracts.

Based on the literature review performed, it can be concluded that DOE methods, because of their appealing advantage to quickly detect how interactions between factors can affect the yield and quality of an obtained extract, are widely applied by researchers. Still, to the best of our knowledge, this work is among the very few to apply a combination of two DOE methods to process optimization and the first case of optimization of the MAE of phenolic compounds from SCGs.

## 2. Results

### 2.1. Fractional Factorial Design (FFD)

In the present study, the effects of four independent parameters (factors) were investigated: microwave power, Power (Watt); ratio between the volumes of the solvent and the solute (SCGs), Ratio Solv/sol (mL/g); composition of the solvent, namely ethanol-to-water ratio, Ratio Eth/water (%Ethanol), and time of microwave irradiation, Time (min). The levels choice was based on the knowledge acquired previously. For a 2^4−1^ (two-level) FFD with four factors, 16 experimental runs were required. It should be noted that, in all the experiments performed, the maximum percentage of water was 50%, since the increase of its content in the final (extract plus solvent) solutions increased considerably the amount of energy required to obtain the final dry extract.

The maximum and minimum of the different factors and their respective coded values generated by the program Design-Expert 11 (DS-11) are shown in Table 1.

The responses characterizing the quality of the extracts in the three response functions are: the yield (%), the total phenolic content, TPC, represented in mg of gallic acid equivalents (GAE) by g of extract (mg GAE/g), and AA as the DPPH assay in micromole of trolox equivalents (TE) by g of extract (µmol TE/g). The results of the FFD design tests are displayed in Table 2.

### 2.2. Statistical Analysis of the FDD Experimental Data

The experimental design applied allowed the identification of the significant experimental factors influencing the yield, TPC, and AA of the extracts recovered. Statistical testing of the model was performed in the form of an analysis of variance (ANOVA) for each response.

The yield, TPC, and AA F-values—65.59, 146.57, and 14.40, respectively—implied that the model was significant. This was corroborated by the values of *p* < 0.0001 and *p* < 0.0006, indicating that there was only a probability of 0.01% for the two first responses and 0.06% for the AA that a higher F-value could occur due to noise. *p*-Values less than 0.05 indicate that the individual model terms are significant.

These results identified the independent variables X_2_—*Ratio (Solv/sol)*—and X_3_—*Ratio (Eth/water)*—as the most significant factors that influence the experimental extraction yield, TPC, and AA, with X_3_ being the dominant one between the two. Thus, for the yield, the contribution percentages of the ratios Solv/sol and Eth/water were 13.52 and 81.44%, respectively, and the combined contribution to the overall model of these two variables was 95%. The same trend was observed concerning TPC and AA, for which the combined contributions were 97.2 and 79.4%, respectively.

The ANOVA results on the FFD model selected are shown in Table 3, showing the most significant terms.

The time (independent variable X_4_), as well as the interaction between X_1_X_2_ (Power * Ratio (Solv/sol)) and X_1_X_4_ (Power*Time), have some significance on AA but with lower key values for the overall model. *p*-Values greater than 0.1000 indicate that the model terms are not significant. Based on the ANOVA analysis and fitting the factors with the responses using the least squares method, the extract yield of the SCGs (Yield), as a function of the independent variables X_1_–X_4_, is obtained by Equation (1).

(1) $$\mathrm{Yield}=-4.32223+0.237833*\mathrm{Ratio}\;\left(\frac{\mathrm{Solv}}{\mathrm{sol}}\right)+0.095561*\mathrm{Ratio}\left(\frac{\mathrm{Eth}}{\mathrm{water}}\right)-0.000986*\mathrm{Power}*\mathrm{Ratio}\left(\frac{\mathrm{Eth}}{\mathrm{water}}\right)+0.000813*\mathrm{Power}*\mathrm{Ratio}\left(\frac{\mathrm{Eth}}{\mathrm{water}}\right)$$

For the TPC (mg GAE/g) and AA (mg GAE/g) of SCG extracts, applying the above methodology, Equations (2) and (3) are obtained, respectively:

(2) $$\mathrm{TPC}=426.49813-3.16167*\mathrm{Ratio}\;\left(\frac{\mathrm{Solv}}{\mathrm{sol}}\right)-2.61480*\mathrm{Ratio}\left(\frac{\mathrm{Eth}}{\mathrm{water}}\right)+0.014444*\mathrm{Power}*\mathrm{Ratio}\left(\frac{\mathrm{Eth}}{\mathrm{water}}\right)-0.002092*\mathrm{Power}*\mathrm{Ratio}\left(\frac{\mathrm{Eth}}{\mathrm{water}}\right)$$

(3) $$\mathrm{DPPH}=188.69866+3.36809*\mathrm{Ratio}\;\left(\frac{\mathrm{Solv}}{\mathrm{sol}}\right)-1.13622*\mathrm{Ratio}\left(\frac{\mathrm{Eth}}{\mathrm{water}}\right)-0.058599*\mathrm{Power}*\mathrm{Ratio}\left(\frac{\mathrm{Eth}}{\mathrm{water}}\right)-0.006550*\mathrm{Power}*\mathrm{Ratio}\left(\frac{\mathrm{Eth}}{\mathrm{water}}\right)$$

The ANOVA attested that a lack of fit (with a *p*-value < 0.0001) was not significant for all response surface models at a 95% confidence level, which means that all models represented the data satisfactorily. Simultaneously, the R^2^, adjusted R^2^ ($R_{\mathrm{adj}}^{2}$), predicted R^2^ ($R_{\mathrm{Pred}}^{2}$), coefficient of variation (CV), and adequate precision (Ad Precision) were calculated to check the model adequacy and are presented in Table 4.

The values of the determination coefficient (R^2^) calculated (98.29, 99.23, and 92.65%) infer that the accuracy and general predictive ability of the quadratic polynomial regression models represented by Equations (1)–(3) are very good, since it is accepted that, for a good fitting model, R^2^ should not be less than 80% [19,20].

Furthermore, the value of the adjusted R^2^ should be close to $R_{\mathrm{adj}}^{2}$, suggesting that a high degree of correlation between the observed and predicted values exists. In addition, $R_{\mathrm{adj}}^{2}$ values should also be in a reasonable agreement with the $R_{\mathrm{Pred}}^{2}$ (differences less than 0.2), showing that nonsignificant terms have not been included in the model.

The coefficient of variation, CV, is a measure expressing the standard deviation as a percentage of the mean. Small values of the CV represent better reproducibility. Overall, a CV higher than 10% signifies that the variation in the mean value is high and does not satisfactorily develop an adequate response model [21].

In our case, the CVs of the yield and TPC are reasonable, while, for the AA, the CV value is the only parameter that does not satisfy the below 10% criterion. Finally, adequate precision (Ad Precision) measures the signal-to-noise ratio, and a value greater than 4 is appropriate, which is verified in all the cases examined (Table 5).

The maximum value of a respective response can be obtained from Equations (1)–(3), respectively. Additionally, if the values corresponding to the simultaneous maximum of the three independent variables—yield, TPC, and AA—are required, those can be obtained by solving the system of Equations (1)–(3). However, as shown, the most important factor for these variations is the ethanol/water ratio, followed by the solvent/solute ratio.

To locate the ideal region of the design space for the chosen experimental factors and intended response, a FFD analysis should be used to assess the main effects and the essential interactions between them. Although Equations (1)–(3) can estimate the fitted values at the corner points of the purpose design, quadratic terms in the model to shape the curvature across the whole response surface are required. That can be achieved by applying a response surface design with axial points generated by a central composite design (CCD).

### 2.3. Central Composite Design (CCD)

The results of the FFD design applied at stage one indicated that the influence of power and time on the MAE was not substantial. Hence, in the CCD, the values of those independent parameters were fixed at 90 W and 4.5 min, respectively.

The other two factors—Ratio Solv/sol (10–20 mL/g) and Ratio Eth/water (50–70% Ethanol)—were used to generate the CCD test design. The ANOVA results on the CCD models selected are shown in Table 5.

It was demonstrated that, for the responses yield and AA, the best fit equation was quadratic, while, for the TPC, a linear one. The F-values showed that the models for the yield, TPC, and AA were significant, and there was only a 0.03, 0.01, and 0.07% chance, respectively, that an F-value that high could occur due to noise. The lack of fit F-values to the three responses was nonsignificant relative to the pure error. The latter was required to have a good model fit and determine the adequacy of the model.

The final models for the responses (yield, TPC, and AA) in terms of actual factors are represented by Equations (4)–(6), respectively.

(4) $$\mathrm{Yield}=14.1922-0.0111515*\mathrm{Ratio}\;\left(\frac{\mathrm{Solv}}{\mathrm{sol}}\right)-0.268848*\mathrm{Ratio}\left(\frac{\mathrm{Eth}}{\mathrm{water}}\right)-0.00415*\mathrm{Ratio}\;\left(\frac{\mathrm{Solv}}{\mathrm{sol}}\right)*\mathrm{Ratio}\left(\frac{\mathrm{Eth}}{\mathrm{water}}\right)+0.01012*\mathrm{Ratio}\;{\left(\frac{\mathrm{Solv}}{\mathrm{sol}}\right)}^{2}+0.002855*\mathrm{Ratio}{\left(\frac{\mathrm{Eth}}{\mathrm{water}}\right)}^{2}$$

(5) $$\mathrm{Polyphenols}=254.87757-0.143982*\mathrm{Ratio}\;\left(\frac{\mathrm{Solv}}{\mathrm{sol}}\right)-1.94491*\mathrm{Ratio}\left(\frac{\mathrm{Eth}}{\mathrm{water}}\right)$$

(6) $$\mathrm{DPPH}=679.41422-23.17815*\mathrm{Ratio}\;\left(\frac{\mathrm{Solv}}{\mathrm{sol}}\right)-9.90032*\mathrm{Ratio}\left(\frac{\mathrm{Eth}}{\mathrm{water}}\right)+0.479841*\mathrm{Ratio}\;{\left(\frac{\mathrm{Solv}}{\mathrm{sol}}\right)}^{2}+0.056789*\mathrm{Ratio}{\left(\frac{\mathrm{Eth}}{\mathrm{water}}\right)}^{2}$$

The ANOVA results on the CCD models selected are shown in Table 6. All terms (independent variables) were significant, since the *p*-values were less than 0.05 (at the 5% probability level), and only values < 0.100 were insignificant.

The ANOVA analysis demonstrated that the models were adequate and reproducible and the results were reliable. Thus, it was ascertained that the regression models obtained were suitable for the determination of significant extraction parameters values that optimized the responses—yield, TPC, and AA—of the SCG extracts recovered.

The response surface analysis was designed centered on the three-dimensional model polynomial function defined, which established the effect of the significant independent variables chosen on each observed response. The contour response plots allowed visual identification of the optimal levels of each factor and a choice of the most suitable values of the different response factors.

**Figure 1:**
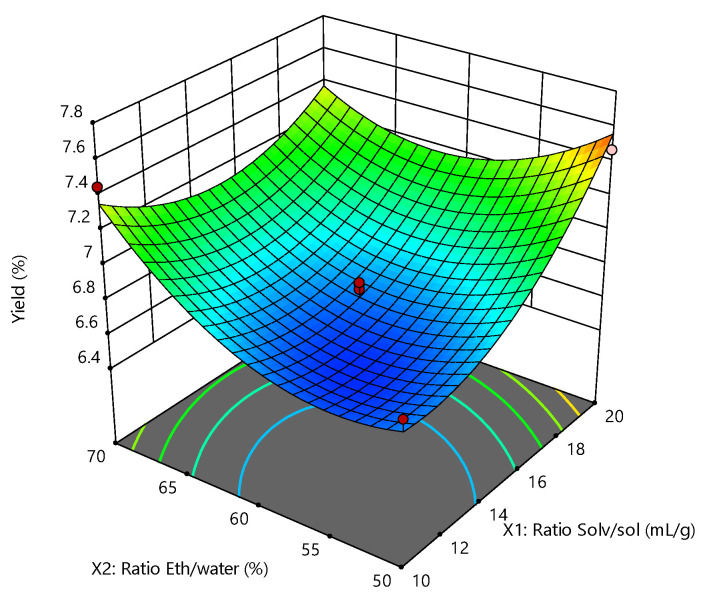
Response surface plot showing the effects of the solvent/solute and ethanol/water ratios on the SCG extraction yield.

It can be deduced from Figure 1 that, when the ratio ethanol/water is kept at a lower level, the yield increases with the growth of the ratio solvent/solute (SCGs). The extraction yield, in terms of a combination of binary factors, can change from 6.58 to 7.64%.

**Figure 2:**
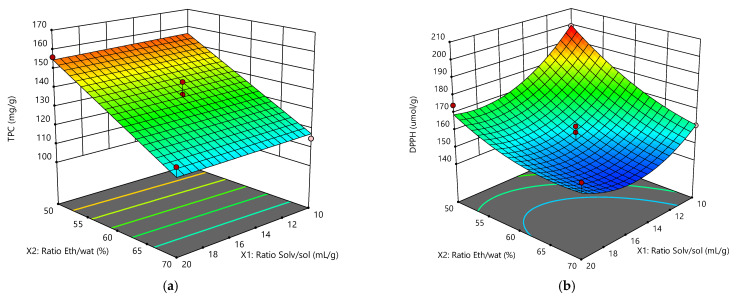
(**a**,**b**) Response surface plots showing the effect of the independent parameters ratios solvent/solute and ethanol/water on TPC and AA of the SCG extracts.

Figure 2a depicts the linear effect of the ratio ethanol/water in the solvent mixture on the TPC. It was demonstrated that, within the entire range of the ratio Solv/sol, the increase of the water percentage in the mixture raised the TPC content. This was in complete agreement with the data of Table 5, where the parameter ratio Eth/water had a *p*-value < 0.0001. On the other hand, the *p*-value of the ratio Solv/sol was not significant, since it was much higher than 0.0001.

Figure 2b shows the quadratic effects of the solvent/solute and ethanol/water ratios on the AA. The maximum was achieved at the lower values of the independent parameters, a behavior pattern completely different from that depicted in Figure 2b.

### 2.4. Optimization of the MAE Process

The target of the response surface methodology is process optimization. Thus, we aimed to determine the values of the two significant independent variables that led to the optimum responses. It should be noted, however, that this was not a trivial task, since their behaviors were divergent, and there was not a single unique point that could be achieved applying Equations (4)–(6). Instead, adjusting a maximum or a minimum to the response, the optimal conditions could be defined randomly, and a few optimal points could be located for them.

To realize that, the DS11 software was applied to determine the best conditions to optimize the three responses. Subsequently, those values were verified by performing three experimental tests to compare them with the optimized ones. The results obtained are presented in Table 7, and they show that the models for predicting the optimum response values were adequate, since there was a very good agreement with the experimental values.

### 2.5. Influence of Ethanol: Water Ratio on the Extracts Composition—^1^H NMR Analysis

In addition to examining the influence of *Ratio (Solv/sol)* and *Ratio (Eth/water)* on the yield, TPC, and AA of the MAE extracts recovered, the second objective of our work was to study the impact of *Ratio (Eth/water)*, identified as the most significant experimental parameter, on the extract’s principal compound compositions. To realize that, an analysis of the extracts obtained in the overall range of the ethanol:water mixture composition employed was carried out. The analyses were performed by applying ^1^H-NMR, which has proven to be a fast and useful tool for composition identification and quantification. The influence of *Ratio (Eth/water)* on the compositions of lipids and diterpenes in the extracts is presented in Table 8 and Table 9, respectively.

Table 8 shows that the % of molar fractions of TAGs decrease smoothly from 96.1 to 85.35%, with the decrease of the percentage of ethanol down to 50%.

The 1,2 DAGs composition, however, exhibited an opposite trend—their % molar fractions increased sharply from 1.96 to 9.04% in the range 99–60% ethanol, reached their maximum at 50% ethanol, and then decreased to 6.53% at 45.9% ethanol.

There was not a clear trend in the change of the composition of the mono- (MUFA) and di-unsaturated fatty acids (DUFA) with the change of the ethanol:water ratio. The only exception was the extract of Run 4, recovered by 99% of ethanol, for which the highest concentration of DUFA (40.4) and the lowest of MUFA (15.5) were recorded.

Table 9 shows the influence of *Ratio (Eth/water)* on the compositions of the diterpenes. The reduction of ethanol % down to 60% positively affected the recovery of the diterpenes. Their % of molar fractions increased, with the highest increment registered in the range of 70–60%, the most pronounced being for cafestol. Interestingly, the diterpene % molar fractions diminished at lower than 60% ethanol in the solvent, and the most significant drop—from 11.12 to 3.17%—was observed for 16-O-Methyl-Cafestol for a relatively small increment from 50 to 45.9% in the ethanol composition. The present results are in good agreement with a previous work, where supercritical CO_2_ was used to obtain the oil.

### 2.6. Influence of Ethanol: Water Ratio on the Eextract Compositions—Fatty Acid Methyl Esters (FAMEs) Analysis

The influence of the ethanol:water ratio on the extracts’ fatty acid compositions was also studied, and the results obtained are presented in Table 10.

The main fatty acids identified in all the samples analyzed were palmitic (C16:0), linoleic (C18:2), oleic (C18:1), and stearic (C18:0) acids. Among those, the most abundant was linoleic acid, its highest quantity registered at 99% of ethanol.

It should be noted, that, unlike the lipids and diterpenes, the fatty acid compositions did not exhibit any clear trends regarding the ethanol:water ratio influence. Still, the highest composition of DUFA (41.8) was registered at the highest value of the ethanol:water ratio, while, for MUFA and SFA, the opposite trend was observed: the highest quantities were registered at the lowest value of that ratio.

As could be expected, the polyunsaturated/saturated ratio UI increased with the increase of the ethanol percentage, and the highest UI was calculated for Run 4, due to the highest content of linoleic acid registered in the extract. The values obtained agreed with the previous results [4,22,23], where the differences in the origin and variety of the coffee and, consequently, the resulting SCGs used could explain some small variations.

## 3. Discussion

There have been few studies published in the literature that have examined alternative techniques for SCG valorization and the recovery of extracts rich in bioactives with pronounced AA and high TPC. The data reported, however, are very scattered and depend on multiple factors, such as the types of solvents, extraction temperatures, solid–liquid ratios, coffee blends and SCGs, and/or different preparation processes, among others, which makes comparisons of the results challenging.

Generally, Soxhlet *n*-hexane extraction is used as the reference method (see, for example, the study of Acevedo et al. [24], where the values of TPC = 273.34 mg GAE/g and DPPH = 82.65 µmol TE/g, respectively, were reported). However, nowadays, green technologies and more environmentally friendly solvents are preferred.

Higher contents of bioactive compounds were registered in SCG extracts of 100% Arabica coffee blends by changing the temperature and the solvent extraction volume at a constant time [25]. The TPC and AA by DPPH of the extracts were 61.49 mg GAE/g and 324.51 µmol TE/g, respectively.

A more comprehensive comparison of our results can be performed with the works of References [12,15], discussed briefly above, who studied the ethanol influence on MAE of SCGs, utilizing RSM. In both works, the % of ethanol in the solvent mixture, however, was different. Ranic et al. [15] applied a fixed percentage of ethanol (20%). The yields achieved were low, but the extracts TPC and AA of the DPPH assay were considerable. On the other hand, Pavlović et al. [12] used mixtures with 20–80% of ethanol. Values of the TPC between 175.08 and 398.95-mg GAE/g, as well as very good DDPH activity (%), were reported.

In view of the above, the most appropriate comparison of our TPC and AA values by DPPH was with those of Pavlović [12].

There were some differences observed in the values of TPC and AA reported by both studies. This was not unexpected, taking into consideration the nature of the SCGs used—filter coffee vs. espresso. Still, our conclusion that the decrease of the % of ethanol in the solvent mixture diminishes the yield of the MAE process while increasing the TPC or DPPH values of the extracts recovered (Table 3 and Table 5) was confirmed and substantiated.

## 4. Materials and Methods

### 4.1. Raw Material

The SCGs, a blended mixture of Arabica and Robusta species, obtained from an espresso machine of a Bulgarian coffee shop were oven-dried to a constant mass at 378 K and stored frozen in a refrigerator at 255 K. The moisture content (4.0 ± 0.3%) was measured with a thermogravimetric balance, Kern MRS 120-3 (KERN & Sohn GmbH, D-72336 Balingen, Germany), and the average particle diameter, dp (0.273 ± 0.023) mm, was calculated as described before [4].

### 4.2. Reagents

Trolox (98%), gallic acid (97.5–102.5%), sodium carbonate anhydrous (Na_2_CO_3_; 99.5%), 2,2-diphenyl-1-picrylhydrazyl (DPPH), and Folin–Ciocalteu reagent, 2 N, were purchased from Sigma Aldrich. Millipore water, ethanol 99%+, absolute, extra pure, SLR, and methanol (99.9%) were from Fisher Chemical.

### 4.3. Microwave Extractions

The microwave-assisted extractions of the SCGs samples were performed using a CEM Discover SP microwave reactor (2.45 GHz, 300 W) (CEM Corporation, Matthews, NC, USA), equipped with a noncontact infrared temperature sensor. The temperature was controlled by the variable microwave irradiations, and the samples were cooled by the nitrogen current at the end of extraction. The microwave radiation ranged from 60 to 120 W and was adjusted to stabilize the temperatures at 75 °C for different extraction times.

For extraction, 1 g of SCGs were placed in a 35-mL pressure vessel containing 15 mL of solvent (a mixture of water–ethanol at different ethanol concentrations (*v*/*v*)). The suspension was irradiated for different periods and at different microwave irradiation powers with stirring. The influence of the solvent mixtures, microwave irradiation powers (60 W and 120 W), irradiation times at the defined temperature (3–6 min), and solvent-to-SCGs ratios on the extraction yield during the extraction process were studied.

The resulting suspension (solvent + extract) was filtered, and the solvent evaporated in a rotary evaporator, Büchi, model R-205 (BUCHI AG, Flawil, Switzerland). The global yield was calculated from the ratio between the mass of extract and the mass of raw material. The extract was stored at −18 °C until use and analyzed for the TPC and DPPH assay.

### 4.4. Antioxidant Activity of DPPH Assay

In our study, the method applied to determine the AA of the extracts was the radical scavenging activity by DPPH. It should be noted that, because the DPPH assay method is usually employed to determine the AA of a sample, a straightforward and direct comparison of the results reported by different authors might be challenging due to dissimilarities in the conditions (reactions and solvents) applied [26].

The DPPH assay of SCG extracts was determined according to the method in the microplates system described previously [27], with modifications. Considering the solvents used in the extractions and the extract solubility, a mixture of ethanol/water (50:50, *v/v*) was used. Thirty microliters of the SCG extracts dissolved in the solvent mixture were set in a microplate (Nunc) with 270 µL of DPPH solution (100 mM) in the same mixture solvents. The solutions were kept at 298 K in the dark, and the absorbance was measured at 517 nm after 40 min in a microplate reader (BioTek Synergy 2, Winooski, VT, USA) in triplicate.

A calibration curve for trolox was used to express the DPPH assay of the extracts as µmol of trolox equivalents by g of extract (µmol TE/g). The inhibition capacity was determined applying Equation (7):

(7) $$IC=\lfloor 1-\left(\frac{A_{s}-A_{b}}{A_{c}-A_{b}})\right\rfloor \times 100$$

where *A_S_*, *A_b_*, and *A_c_* are the measured absorbance of the sample, the blank with pure solvent, and the control with the solution of DPPH, respectively. All measurements were done in triplicate.

The analysis was done by linear regression and using ANOVA [28], presented in Equation (8), where *C_S_* is the concentration of the sample in µg/mL, with an R^2^ = 0.998. The inhibition concentration to trolox at 50% was IC_50_ = 46.60 ± 1.19 µg/mL. The sensitivity was determined according to the limit of detection (LOD = 2.35 µg/mL) and limit of quantification (LOQ = 7.83 µg/mL) [29].

(8) $$A_{s}=-5.100\;\left(\pm \;2.270\right)+1.180\left(\pm \;0.045\right)C_{s}$$

### 4.5. Total Polyphenol Content

The total polyphenol content of the SCG extracts was determined quantitatively using the Folin–Ciocalteu reagent in a microplates system with modifications [27]. In a microplate (Nunc), 30 μL of the extracts (previously dissolved in ethanol/water, 50/50 (*v*/*v*)) were mixed with 150 μL of Folin–Ciocalteu reagent, prepared from a 2-N solution, and diluted in distilled water (1:10, *v*/*v*). After standing for 4 min, 120 μL of a solution of Na_2_CO_3_ (75 g/L) were added, the mixture was left at 40 °C for 30 min, and the absorbance was measured at 765 nm in a microplate reader (BioTek Synergy 2, Winooski, VT, USA) in triplicate. The TPC was expressed as mg of gallic acid equivalents (GAE) by g of extract (mg GAE/g), determined from a calibration curve.

The calibration curve of gallic acid was built following the method described above to the samples in triplicate, and the results are represented by Equation (9), with an R^2^ = 0.991. The sensitivity was determined according to the limit of detection (LOD = 2.92 µg) and limit of quantification (LOQ = 9.75 µg).

(9) $$A_{s}=0.00901\;\left(\pm \;0.00180\right)+0.02695\left(\pm \;0.00081\right)C_{s}$$

### 4.6. Analysis of the Extracts by ^1^H NMR

The analysis was performed according to the method described before [4], with the necessary adaptations. Briefly, samples of 0.015–0.025 g of extracts dissolved in 500 μL 75–100-mM solutions of CDCl_3_ were used for recording the proton NMR spectra. The chemical shifts (δ) for the different components were assigned based on the values reported in the literature for TAGs and 1,2-DAGs [30], caffeine [31], cafestol, 16-O-methylcafestol [32], and kahweol [33].

### 4.7. Analysis of the Fatty Acid Methyl Esters (FAMEs)

A gas chromatographic method was developed to characterize the fatty acid ester profiles of the SCG extracts achieved. The assessments were done regarding the parameters in Annex I to Commission Regulation (EEC) No. 2568/91(1), CELEX_01991R2568 published 12 April 2016, with the required adjustments. The transesterification of the extracts into fatty acid methyl esters (FAMEs) was performed on a methanol solution of KOH (2M).

### 4.8. Design of Experiments

Design of experiments is a fundamental tool for research and development, which provides a way of analyzing areas where the basic knowledge is minimal and needs to be constructed methodically and successfully.

We used a 2^4−1^ two-level Fractional Factorial Design with four factors on stage one. In a two-level (2-level) FFD, each experimental factor has only two levels, and the experimental runs include all combinations of these factors. Although 2-level factorial designs are unable to fully explore a wide region in the factor space, they provide useful information for relatively few runs per factor.

Once the most significant independent parameters (factors) identified (ethanol:water ratio and solid/solute ratio) in a stage two Central Composite Design (CCD) was used to examine the effects of those variables concerning their responses and quadratic surfaces to optimize the values with a minimum number of experiments generated [34,35].

An empirical model that correlated the response to the independent parameters using a polynomial equation, like the one given by Equation (10), was applied:

(10) $$Y=a_{0}+\overset{n}{\underset{i=1}{\sum }}a_{i}X_{i}+{\left(\overset{n}{\underset{i=1}{\sum }}a_{\mathrm{ii}}X_{i}\right)}^{2}+\overset{n-1}{\underset{i=1}{\sum }}\overset{n}{\underset{j=i+1}{\sum }}a_{\mathrm{ij}}X_{i}X_{j}$$

where *Y* is the predicted response, a_0_—the constant coefficient, a_i_—the linear coefficients, a_ii_—the quadratic coefficients, a_ij_—the interaction coefficients, and X_i_ and X_j_ are the coded values of the independent parameters in the experience.

## 5. Conclusions

In this work, two DOE methods—a FFD followed by a CCD—were successfully implemented for the first time in the optimization of MAE of SCGs in terms of the extraction yield, TPC, and AA as the DDPP assay. Furthermore, the composition and fatty acid profile of the extracts recovered were analyzed by NMR and GC-FID.

It was demonstrated that the influence of the factor of the principal significance—*Ratio (Eth/water)*—on the extraction process responses of yield, TPC, and AA was substantial. While low ratio values (decrease of ethanol percent in the solvent mixture) decreased considerably the yield, it positively affected the TPC and AA of the extracts obtained. The significant impact of the variations of the % of ethanol in the solvent mixture on the compositions of the lipids, caffeine, pentacyclic diterpenes of the kaurene family, and FAME was also confirmed by the NMR and GC-FID analyses of the extracts recovered.

## Figures and Tables

**Table 1:** Range of parameters examined.

Factor	Name	Units	Coded Low	Coded High
X_1_	Power	Watt	−1 ≡ 60.00	+1 ≡ 120.00
X_2_	Ratio (Solv/sol)	mL/g	−1 ≡ 5.00	+1 ≡ 20.00
X_3_	Ratio (Eth/water)	% Ethanol	−1 ≡ 50.00	+1 ≡ 99.00
X_4_	Time	min	−1 ≡ 3.00	+1 ≡ 6.00

**Table 2:** FFD design tests results and the response functions evaluated in each experiment.

	X_1_	X_2_	X_3_	X_4_	Resp. 1	Resp. 2	Resp. 3
Run	Power	Ratio (Solv/sol)	Ratio (Eth/water)	Time	Yield	TPC	AA
	Watt	mL/g	%Ethanol	min	%	mg GAE/g	µmol TE/g
1	120	5	50	6	4.5	210.7 ± 11.7	185.3 ± 8.3
2	60	5	50	3	4.84	202.8 ± 10.9	150.9 ± 7.7
3	60	20	99	3	12.96	35.0 ± 1.4	85.3 ± 2.8
4	120	20	99	6	12.52	41.9 ± 2.5	66.4 ± 3.4
5	120	5	99	3	11.12	44.1 ± 1.3	62.0 ± 1.7
6	60	5	50	3	4.54	209.8 ± 8.2	147.7 ± 4.8
7	120	5	99	3	10.7	58.1 ± 6.2	87.9 ± 2.7
8	120	20	50	3	7.39	169.5 ± 10.2	95.2 ± 7.2
9	120	20	50	3	6.5	170.7 ± 6.6	133.2 ± 4.5
10	120	20	99	6	12.88	42.1 ± 3.8	37.4 ± 2.3
11	60	20	50	6	8.02	153.0 ± 5.2	148 ± 5.8
12	60	20	99	3	12.22	40.2 ± 3.4	59.0 ± 3.4
13	60	5	99	6	11.23	41.5 ± 0.9	97.6 ± 9.7
14	60	20	50	6	9.3	144.2 ± 4.0	158.8 ± 5.2
15	60	5	99	6	11.17	56.0 ± 5.3	63.8 ± 2.5
16	120	5	50	6	5.8	190.7 ± 6.3	216.7 ± 5.4

**Table 3:** ANOVA results on the FFD model selected.

	Yield (%)				TPC (mg GAE/g)				AA (µmol TE/g)			
Source	SS ^a^	MS ^b^	F-Value	*p-*Value	SS ^a^	MS ^b^	F-Value	*p-*Value	SS ^a^	MS ^b^	F-Value	*p-*Value
Model	145.43	20.78	65.59	<0.0001	80,339.8	11,477.1	146.57	<0.0001	2329.14	332.73	14.4	0.0006
X_1_-Power	0.5148	0.5148	1.63	0.2381	166.41	166.41	2.13	0.183	2.87	2.87	0.124	0.7335
X_2_-Ratio (Solv/sol)	20	20	63.16	<0.0001	3119.22	3119.22	39.83	0.0002	204.78	204.78	8.86	0.0177
X_3_-Ratio (Eth/water)	120.51	120.51	380.47	<0.0001	75,460.1	75,460.1	963.65	<0.0001	1791.83	1791.83	77.54	<0.0001
X_4_-Time	1.66	1.66	5.23	0.0514	119.9	119.9	1.53	0.251	91.3	91.3	3.95	0.0821
X_1_X_2_	0.7877	0.7877	2.49	0.1535	169	169	2.16	0.18	174.24	174.24	7.54	0.0252
X_1_X_3_	0.2889	0.2889	0.9121	0.3675	37.82	37.82	0.483	0.5067	23.23	23.23	1.01	0.3454
X_1_X_4_	1.67	1.67	5.27	0.0507	1267.36	1267.36	16.18	0.0038	40.9	40.9	1.77	0.2201
Pure Error	2.53	0.3167			626.45	78.31			184.87	23.11		
Cor Total	147.96				80,966.3				2514.01			

^a^ Sums of squares. ^b^ Mean square.

**Table 4:** Analysis of variance for the fitted models (Fit statistics).

Fit Statistics	Yield (%)	TPC (mg GAE/g)	AA (µmol TE/g)
R^2^	0.9829	0.9923	0.9265
Adjusted R^2^	0.9679	0.9855	0.8621
Predicted R^2^	0.9315	0.9691	0.7059
CV (%)	6.18	7.79	17.12
Ad Precision	20.1281	26.9607	10.9821

**Table 5:** CCD test design results and the response evaluated in each experiment.

Run	Type	X_1_	X_2_	Resp. 1	Resp. 2	Resp. 3
		Ratio (Solv/sol)	Ratio (Eth/water)	Yield	TPC	AA
		mL/g	%Ethanol	%	mg GAE/g	µmol TE/g
1	Axial	15	45.9	7.14	161.1 ± 3.7	181.7 ± 6.8
2	Axial	15	74.1	7.43	103.9 ± 8.7	142.6 ± 12.2
3	Factorial	20	70	7.31	120.7 ± 6.9	158.9 ± 4.4
4	Center	15	60	6.8	143.8 ± 5.3	161.2 ± 2.4
5	Center	15	60	6.76	137.2 ± 6.2	157.7 ± 5.5
6	Center	15	60	6.64	131.0 ± 5.1	150.9 ± 7.0
7	Center	15	60	6.58	132.1 ± 8.1	150.9 ± 7.8
8	Factorial	20	50	7.47	156.0 ± 6.7	174.5 ± 10.5
9	Factorial	10	50	6.78	154.0 ±10.3	199.2 ± 3.8
10	Axial	7.9	60	6.8	143.9 ± 2.8	192.4 ± 7.7
11	Center	15	60	6.79	135.9 ± 12.1	145.4 ± 10.4
12	Factorial	10	70	7.45	114.6 ± 2.5	160.7 ± 8.9
13	Axial	22.1	60	7.64	134.1 ± 10.1	157.2 ± 4.3

**Table 6:** ANOVA results on the CCD models selected. Estimated regression model of the relationship between a response variable and the independent variables.

	Yield (%)				TPC (mg GAE/g)				AA (µmol TE/g)			
Source	SS ^a^	MS ^b^	F-Value	*p-*Value	SS ^a^	MS ^b^	F-Value	*p-*Value	SS ^a^	MS ^b^	F-Value	*p-*Value
Model	1.55	0.3104	23.92	0.0003	3030.29	1515.2	70.57	<0.0001	3477.46	695.49	17.86	0.0007
X1-Ratio (Solv/sol)	0.3776	0.3776	29.09	0.001	4.15	4.15	0.1931	0.6697	729.64	729.64	18.73	0.0034
X_2_-Ratio (Eth/water)	0.1058	0.1058	8.16	0.0245	3026.15	3026.2	140.96	<0.0001	1495.73	1495.73	38.4	0.0004
X_1_X_2_	0.1722	0.1722	13.27	0.0083					131.21	131.21	3.37	0.1091
X_1_^2^	0.4453	0.4453	34.31	0.0006					1001.07	1001.07	25.7	0.0014
X_2_^2^	0.567	0.567	43.7	0.0003					224.35	224.35	5.76	0.0475
Residual	0.0908	0.013			214.69	21.47			272.63	38.95		
Lack of Fit	0.0521	0.0174	1.79	0.2875	112.19	18.7	0.7297	0.6529	115.73	38.58	0.9835	0.4847
Pure Error	0.0387	0.0097			102.5	25.62			156.9	39.23		
Cor Total	1.64				3244.98				3750.09			

^a^ Sums of squares. ^b^ Mean square.

**Table 7:** Predicted and experimental values of the responses were obtained at the optimum conditions of the independent variables. The experimental data are given as the mean ± SD (*n* = 3).

	X_1_-Ratio (Solv/sol)	X_2_-Ratio (Eth/Water)	Yields (%)	TPC (mg GAE/g)	DPPH (µmol TE/g)
Predicted values	16.6757	68.862	7.07974	118.546	145.413
Experimental values	16.7	68.9	6.98 ± 0.27	117.7 ± 6.1	143.8 ± 8.6

**Table 8:** Lipids compositions of the SCG extracts, obtained by the MAE, as established by an ^1^H-NMR quantitative analysis. All values represent the % of molar fractions. The unsaturation index (UI) is defined by UI = (2 × DUFA % molar fraction + MUFA % molar fraction)/100.

	CCD					FFD
	Run 1	Run 8	Run 5	Run 12	Run 2	Run 4
Lipids (%_mol_)	%EtOH (45.9)	%EtOH (50)	%EtOH (60)	%EtOH (70%)	%EtOH (74.1)	%EtOH (99)
TAG	92.66	85.35	88.13	89.9	92.5	96.1
1,2 DAG	6.52	9.66	9.04	6.98	3.45	1.89
Caffeine	3.33	4.98	2.83	3.12	2.73	2.01
DUFA	34.0	34.5	35.1	35.7	34.5	40.4
MUFA	21.5	23.3	19.2	19.6	22.3	15.5
SFA	44.5	42.7	45.8	44.7	43.3	44.1
UI	0.895	0.922	0.893	0.91	0.912	0.963

**Table 9:** Diterpene contents of the SCG extracts, obtained by MAE established quantitatively by ^1^H-NMR.

	CCD					FFD
	Run 1	Run 8	Run 5	Run 12	Run 2	Run 4
Compounds (%_mol_)	%EtOH (45.9)	%EtOH (50)	%EtOH (60)	%EtOH (70%)	%EtOH (74.1)	%EtOH (99)
Cafestol	11.29	14.45	17.2	9.83	7.89	6.32
16-O-Methyl-Cafestol	3.17	11.12	12.49	10.27	9.23	4.37
Kahweol	1.76	3.66	4.8	2.74	2.05	1.98
Diterpene content (%)	16.22	29.24	34.49	22.84	20.6	12.67

**Table 10:** Compositions of the esters of fatty acids obtained with MAE, % mass *.

	CCD					FFD
	Run 1	Run 8	Run 5	Run 12	Run 2	Run 4
Fatty Acid Ester	%EtOH (45.9)	%EtOH (50)	%EtOH (60)	%EtOH (70%)	%EtOH (74.1)	EtOH (99)
C12:0—Lauric	0	0	0	0	0	0
C14:0—Myristic	0.06	0.09	0.09	0.05	0.09	0.09
C16:0—Palmitic	33.6	32.22	32.97	32.75	32.64	33.1
C16:1—Palmitoleic	0.22	0.12	0.1	0.12	0.11	0.11
C18:0—Stearic	7.09	7.18	7.32	7.18	7.21	7.11
C18:1—Oleic	16.62	16.9	16.31	16.34	16.52	12.62
C18:2—Linoleic	36.01	37.1	38.08	38.22	37.51	41.48
C18:3—Linolenic	1.2	1.47	0.85	0.9	0.85	0.78
C20:0—Arachidic	2.86	2.59	2.54	2.73	3.01	3.01
C20:1—Gadoleic	0.63	0.28	0.39	0.37	0.48	0.4
C22:0 —Behenic	0.5	0.44	0.39	0.36	0.46	0.36
C22:1—Erucic	0.02	0	0	0	0	0
C24:0—Lignoceric	0	0.13	0.16	0.1	0.13	0.13
C24:1—Nervonic	0	0	0	0.01	0.02	0.02
DUFA	36.01	37.1	38.08	38.22	37.51	41.48
MUFA	17.49	17.3	16.8	16.84	17.13	13.15
SFA	44.11	42.65	43.47	43.18	43.56	43.82
UI	0.895	0.915	0.930	0.933	0.922	0.961

* The lowest limit under which concentrations could not be determined quantitatively by the method used was 0.03. These concentrations were assumed to be 0.00 %_mass._

## Data Availability

Not applicable.

## Sample Availability

Samples of the SCGs and/or extracts are available from the authors.

## References

[1] The International Coffee Organization. https://www.ico.org/.

[2] Georgieva S.S., Coelho J.A.P., Campos F.C., Robalo M.P., Stateva R.P. (2018). Green extraction of high added value substances from spent coffee grounds: Preliminary results. J. Chem. Technol. Metall..

[3] Karmee S.K. (2018). A spent coffee grounds based biorefinery for the production of biofuels, biopolymers, antioxidants and biocomposites. Waste Manag..

[4] Coelho J.P., Filipe R.M., Paula Robalo M., Boyadzhieva S., Cholakov G.S., Stateva R.P. (2020). Supercritical CO_2_ extraction of spent coffee grounds. Influence of co-solvents and characterization of the extracts. J. Supercrit. Fluids.

[5] Routray W., Orsat V. (2012). Microwave-Assisted Extraction of Flavonoids: A Review. Food Bioprocess Technol..

[6] Ameer K., Shahbaz H.M., Kwon J.H. (2017). Green Extraction Methods for Polyphenols from Plant Matrices and Their Byproducts: A Review. Compr. Rev. Food Sci. Food Saf..

[7] Cardoso-Ugarte G.A., Juárez-Becerra G.P., Sosa-Morales M.E., López-Malo A. (2013). Microwave-assisted extraction of essential oils from herbs. J. Microw. Power Electromagn. Energy.

[8] Tzanova M., Atanasov V., Yaneva Z., Ivanova D., Dinev T. (2020). Selectivity of current extraction techniques for flavonoids from plant materials. Processes.

[9] Banožić M., Banjari I., Flanjak I., Paštar M., Vladić J., Jokić S. (2021). Optimization of MAE for the separation of nicotine and phenolics from tobacco waste by using the response surface methodology approach. Molecules.

[10] Chaves J.O., de Souza M.C., da Silva L.C., Lachos-Perez D., Torres-Mayanga P.C., da Fonseca Machado A.P., Forster-Carneiro T., Vázquez-Espinosa M., González-de-Peredo A.V., Barbero G.F. (2020). Extraction of Flavonoids From Natural Sources Using Modern Techniques. Front. Chem..

[11] Chandrasekaran S., Ramanathan S., Basak T. (2013). Microwave food processing-A review. Food Res. Int..

[12] Pavlović M.D., Buntić A.V., Šiler-Marinković S.S., Dimitrijević-Branković S.I. (2013). Ethanol influenced fast microwave-assisted extraction for natural antioxidants obtaining from spent filter coffee. Sep. Purif. Technol..

[13] Passos C.P., Coimbra M.A. (2013). Microwave superheated water extraction of polysaccharides from spent coffee grounds. Carbohydr. Polym..

[14] Passos C.P., Sério A., Ferreira S.S., Kukurová K., Ciesarová Z., Nunes F.M., Coimbra M.A. (2015). Microwave assisted extraction of carbohydrate-rich fractions from spent coffee grounds: Formulation of biscuits enriched in dietary fibre. Trends Carbohydr. Res..

[15] Ranic M., Nikolic M., Pavlovic M., Buntic A., Siler-Marinkovic S., Dimitrijevic-Brankovic S. (2014). Optimization of microwave-assisted extraction of natural antioxidants from spent espresso coffee grounds by response surface methodology. J. Clean. Prod..

[16] Pettinato M., Alberto A., Perego P. (2019). Food and Bioproducts Processing The role of heating step in microwave-assisted extraction of polyphenols from spent coffee grounds. Food Bioprod. Process..

[17] Hibbert S., Welham K., Zein S.H. (2019). An innovative method of extraction of coffee oil using an advanced microwave system: In comparison with conventional Soxhlet extraction method. SN Appl. Sci..

[18] Pettinato M., Casazza A.A., Ferrari P.F., Palombo D., Perego P. (2019). Eco-sustainable recovery of antioxidants from spent coffee grounds by microwave-assisted extraction: Process optimization, kinetic modeling and biological validation. Food Bioprod. Process..

[19] Karazhiyan H., Razavi S.M.A., Phillips G.O. (2011). Extraction optimization of a hydrocolloid extract from cress seed (Lepidium sativum) using response surface methodology. Food Hydrocoll..

[20] Chen W., Wang W.P., Zhang H.S., Huang Q. (2012). Optimization of ultrasonic-assisted extraction of water-soluble polysaccharides from Boletus edulis mycelia using response surface methodology. Carbohydr. Polym..

[21] Myers R.H., Montgomery D.C., Anderson-Cook C.M., Balding D.J., Cressie N.A.C., Fitzmaurice G.M., Givens G.H., Goldstein H., Molenberghs G., Scott D.W., Smith A.F.M., Tsay R.S., Weisberg S. (2016). Response Surface Methodology: Process and Product Optimization Using Designed Experiments.

[22] Couto R.M., Fernandes J., da Silva M.D.R.G., Simões P.C. (2009). Supercritical fluid extraction of lipids from spent coffee grounds. J. Supercrit. Fluids.

[23] Boyadzhieva S., Angelov G., Georgieva S., Yankov D. (2018). Characterization of polyphenol content and antioxidant capacity of spent coffee grounds. Bulg. Chem. Commun..

[24] Acevedo F., Rubilar M., Scheuermann E., Cancino B., Uquiche E., Garcés M., Inostroza K., Shene C. (2013). Spent coffee grounds as a renewable source of bioactive compounds. J. Biobased Mater. Bioenergy.

[25] Gigliobianco M.R., Campisi B., Peregrina D.V., Censi R., Khamitova G., Angeloni S., Caprioli G., Zannotti M., Ferraro S., Giovannetti R. (2020). Optimization of the extraction from spent coffee grounds using the desirability approach. Antioxidants.

[26] Sharma O.P., Bhat T.K. (2009). DPPH antioxidant assay revisited. Food Chem..

[27] Bobo-García G., Davidov-Pardo G., Arroqui C., Marín-Arroyo M.R., Vírseda P., Marín-Arroyo M.R., Navarro M. (2015). Intra-laboratory validation of microplate methods for total phenolic content and antioxidant activity on polyphenolic extracts, and comparison with conventional spectrophotometric methods. J. Sci. Food Agric..

[28] Miller J., Miller J. (2005). Statistics and Chemometrics for Analytical Chemistry.

[29] Shrivastava A., Gupta V.B. (2011). Methods for the determination of limit of detection and limit of quantitation of the analytical methods. Chron. Young Sci..

[30] Fiori L., Lavelli V., Duba K.S., Sri Harsha P.S.C., Mohamed H.B., Guella G. (2014). Supercritical CO_2_ extraction of oil from seeds of six grape cultivars: Modeling of mass transfer kinetics and evaluation of lipid profiles and tocol contents. J. Supercrit. Fluids.

[31] Monakhova Y.B., Ruge W., Kuballa T., Ilse M., Winkelmann O., Diehl B., Thomas F., Lachenmeier D.W. (2015). Rapid approach to identify the presence of Arabica and Robusta species in coffee using 1H NMR spectroscopy. Food Chem..

[32] Guercia E., Berti F., Navarini L., Demitri N., Forzato C. (2016). Isolation and characterization of major diterpenes from C. canephora roasted coffee oil. Tetrahedron Asymmetry.

[33] Scharnhop H., Winterhalter P. (2009). Isolation of coffee diterpenes by means of high-speed countercurrent chromatography. J. Food Compos. Anal..

[34] Khuri A.I., Cornell J.A. (2018). Response Surface, Design and Analysis.

[35] Santos C.P., Rato T.J., Reis M.S. (2019). Design of Experiments: A comparison study from the non-expert user’s perspective. J. Chemom..

